# The relationship between long noncoding RNA *H19* genotypes and the clinical features of diabetic retinopathy

**DOI:** 10.7150/ijms.105022

**Published:** 2025-01-01

**Authors:** Michael Chia-Yen Chou, Hsiang-Wen Chien, Chia-Yi Lee, Shun-Fa Yang, Hung-Yu Lin

**Affiliations:** 1 Institute of Medicine, Chung Shan Medical University, Taichung, Taiwan.; 2 Department of Ophthalmology, Show Chwan Memorial Hospital, Changhua, Taiwan.; 3 Department of Ophthalmology, Cathay General Hospital, Taipei, Taiwan.; 4 Departments of Ophthalmology, Sijhih Cathay General Hospital, New Taipei City, Taiwan.; 5 School of Medicine, National Tsing Hua University, Hsinchu, Taiwan.; 6 School of Medicine, College of Medicine, Fu Jen Catholic University, New Taipei, Taiwan.; 7 Department of Medical Research, Chung Shan Medical University Hospital, Taichung, Taiwan.; 8 Department of Post-Baccalaureate Medicine, College of Medicine, National Chung Hsing University, Taichung, Taiwan.; 9 Department of Optometry, Chung Shan Medical University, Taichung, Taiwan.

**Keywords:** diabetic retinopathy, H19, single-nucleotide polymorphisms, glomerular filtration rate, age

## Abstract

Diabetic retinopathy (DR) is a microvascular complication of diabetes characterized by an inflammatory response. The H19 gene plays a role in regulating inflammation and is associated with chronic systemic inflammation. This study aims to investigate the potential correlation between single-nucleotide polymorphisms (SNPs) in the *H19* gene and the development of DR. Five loci of *H19* SNPs—rs3024270 (C/G), rs2839698 (C/T), rs3741219 (A/G), rs2107425 (C/T), and rs217727 (C/T)—were genotyped using TaqMan allelic discrimination in 454 individuals without DR and 272 DR participants. The results indicate that the *H19* SNP rs3741219 AG (p = 0.030) and AG+GG (p = 0.037) alleles are significantly associated with an increased risk of developing DR in individuals with diabetes onset before the age of 45. Additionally, diabetic individuals with the *H19* SNP rs3741219 AG+GG genotype also showed significantly higher serum creatinine (p = 0.034), lower glomerular filtration rate (GFR) (p = 0.013), higher total cholesterol/HDL ratio (p = 0.031), and higher triglycerides (p = 0.012). In an age-based subgroup analysis, GFR was significantly lower in diabetic patients with an onset of diabetes before 45 years and with the *H19* SNP rs3741219 AG+GG genotype (p = 0.012). In conclusion, the presence of the *H19* SNP rs3741219 variant is associated with a higher risk of DR in individuals with early-onset diabetes, and the relationship between the rs3741219 variant and decreased GFR is particularly pronounced in this population.

## Introduction

Diabetes mellitus is a widespread disease characterized by insulin deficiency, pancreatic β-cell dysfunction, and insulin resistance in the human body 1. It can lead to several vascular complications, one of which is diabetic retinopathy (DR), a condition with high prevalence and an annual incidence exceeding 10 percent among diabetic patients 2-4. In Asia, the risk of DR and DR-related visual impairment has increased in recent years, and advanced DR can result in significant visual disability 5, 6. The most critical and foundational treatment for DR is the management of hyperglycemia, while intravitreal injection of anti-vascular endothelial growth factor (anti-VEGF) and panretinal photocoagulation may be necessary for severe cases of DR 7.

Certain parameters have been correlated with the development of DR in previous publications 8. Hyperglycemia and elevated glycated hemoglobin (HbA1c) concentrations are well-established indicators for the development of DR in individuals with diabetes 5, 9. Specifically, the incidence of DR increases by 15 percent for each 1 percent rise in HbA1c levels 8. Additionally, dyslipidemia, including elevated serum LDL and triglyceride levels, has also been associated with the development of DR in earlier studies 10. Inflammatory biomarkers, such as interleukin and C-reactive protein, are predictors for the onset of DR 11. Regarding the genetic aspect, single nucleotide polymorphisms (SNPs) in the *CDKN2B-AS1*, *MEG3*, and *GAS5* genes have been linked to a higher incidence of advanced DR 12-14.

The H19 gene is a long noncoding RNA that regulates cell proliferation and tumorigenesis in humans 15-18. The presence of the *H19* gene has been associated with an increased risk of colorectal and gastric cancers 16, while polymorphisms in the *H19* gene have been shown to influence the incidence of bladder cancer 19.

However, the relationship between *H19* gene polymorphisms and the risk of DR remains unclear. Given that the H19 gene is also involved in inflammation, a key pathophysiological factor in DR 5, 20, a potential correlation between them may exist.

Therefore, the objective of the present study is to evaluate the potential association between *H19* gene SNPs and the clinical manifestations of DR in diabetic patients. A subgroup analysis will be conducted to examine patients with different ages separately.

## Materials and Methods

### Ethic declarations

All interventions in the present study adhered to the Declaration of Helsinki (1964) and its subsequent amendments. The study was approved by the Institutional Review Board of Chung Shan Medical University Hospital (project identification code: CS2-22190). Written informed consent was obtained from all participants in the study.

### Participant selection

A total of 726 diabetic participants were recruited from Chung Shan Medical University Hospital in Taichung, Taiwan. Of these, 454 participants were assigned to the non-DR group, and 272 participants to the DR group. Medical records for all participants were reviewed, and the presence of DR was defined by the existence of any of the following ophthalmic conditions: dot or flame-shaped hemorrhages, microaneurysms, hard exudates, intraretinal microvascular abnormalities, cotton-wool spots, venous beading, or retinal neovascularization. Among the study population, 223 participants were aged 45 years or younger, and 503 participants were older than 45 years.

### Medical profiles and samples obtainment

Medical information, including age at DR onset, sex, diabetes duration, HbA1c level, serum lipid concentrations, and kidney function (specifically serum creatinine and glomerular filtration rate, or GFR), was collected. For the H19 gene and genetic polymorphism analysis, blood samples were drawn from the vein of each participant and stored in tubes containing ethylenediaminetetraacetic acid (EDTA). The samples were then centrifuged and stored in a special freezer at -80°C. If a participant's genomic DNA or blood sample degraded before DNA analysis, that participant was excluded from the study.

### DNA determination of H19 SNPs by Real-Time PCR

Five *H19* SNPs, including rs3024270 (C/G), rs2839698 (C/T), rs3741219 (A/G), rs2107425 (C/T), and rs217727 (C/T), were selected based on the fact that the genotype frequencies of the minor alleles of these SNPs were greater than 5%, and because previous studies have linked these SNPs to the occurrence of specific diseases 21-25. The DNA extraction procedures in this study followed the methods described in a previous publication 26. Genomic DNA from leukocytes in venous blood samples was extracted using QIAamp DNA kits (Qiagen, Valencia, CA, USA) according to the manufacturer's instructions. The isolated DNA was stored at -20°C. The polymorphisms of the *H19* SNPs (rs3024270, assay ID: C_15833426_10; rs2839698, assay ID: C_2603701_10; rs3741219, assay ID: C_27492510_10; rs2107425, assay ID: C_16032886_10; rs217727, assay ID: C_2603707_10) were subsequently analyzed using the ABI StepOne Real-Time PCR System (Applied Biosystems, Foster City, CA, USA), and the results were further processed using SDS version 3.0 software (Applied Biosystems, Foster City, CA, USA).

### Statistical analysis

Statistical analyses in the present study were conducted using SAS version 9.4 (SAS Institute Inc., NC, USA). The independent t-test and Chi-square test were used to assess differences in baseline characteristics between the two groups. Subsequently, multiple logistic regression analysis was performed to estimate the adjusted odds ratios (AORs) with 95% confidence intervals (CIs) for the frequencies of five SNPs between the non-DR and DR groups. The multiple logistic regression model accounted for potential confounders, including age, duration of diabetes, HbA1c levels, serum creatinine, glomerular filtration rate (GFR), HDL cholesterol, and the total cholesterol/HDL ratio. A p-value of less than 0.05 was considered statistically significant.

## Results

### Initial features between the non-DR group and DR group

The baseline characteristics of the two groups are presented in Table 1. The mean age was significantly lower in the non-DR group (60.22 ± 11.22 years) compared to the DR group (62.59 ± 10.79 years; p = 0.005). However, there was no significant difference in the age of diabetes onset between the two groups (50.84 ± 10.78 years versus 50.59 ± 11.17 years; p = 0.767), and the sex distribution did not differ significantly between the groups (p = 0.550). Regarding laboratory data, the DR group exhibited significantly longer duration of diabetes, higher HbA1c levels, higher serum creatinine, lower glomerular filtration rate (GFR), lower HDL cholesterol, and a lower total cholesterol/HDL ratio compared to the non-DR group (all p < 0.05) (Table 1).

### The genotype frequency of H19 SNP between the non-DR group and DR group

Regarding the distribution of *H19* SNP genotypes between the non-DR and DR populations, none of the five *H19* SNPs showed a significant association with the development of DR (all p > 0.05) (Table 2). In the age-based subgroup analysis, the *H19* SNP rs3741219 AG genotype (AOR: 1.888, 95% CI: 1.065-3.348, p = 0.030) and the AG+GG genotype combination (AOR: 1.778, 95% CI: 1.034-3.057, p = 0.037) were significantly associated with an increased risk of DR development in individuals with diabetes onset before the age of 45 (Table 3). In contrast, no other *H19* SNPs showed a significant association with DR development in this age subgroup (all p > 0.05). In the subgroup of individuals with diabetes onset after the age of 45, none of the *H19* SNPs were significantly associated with an increased risk of DR (all p > 0.05) (Table 3).

### Correlation between the clinical features and the H19 SNP rs3741219 genotypes

**Figure 1:**
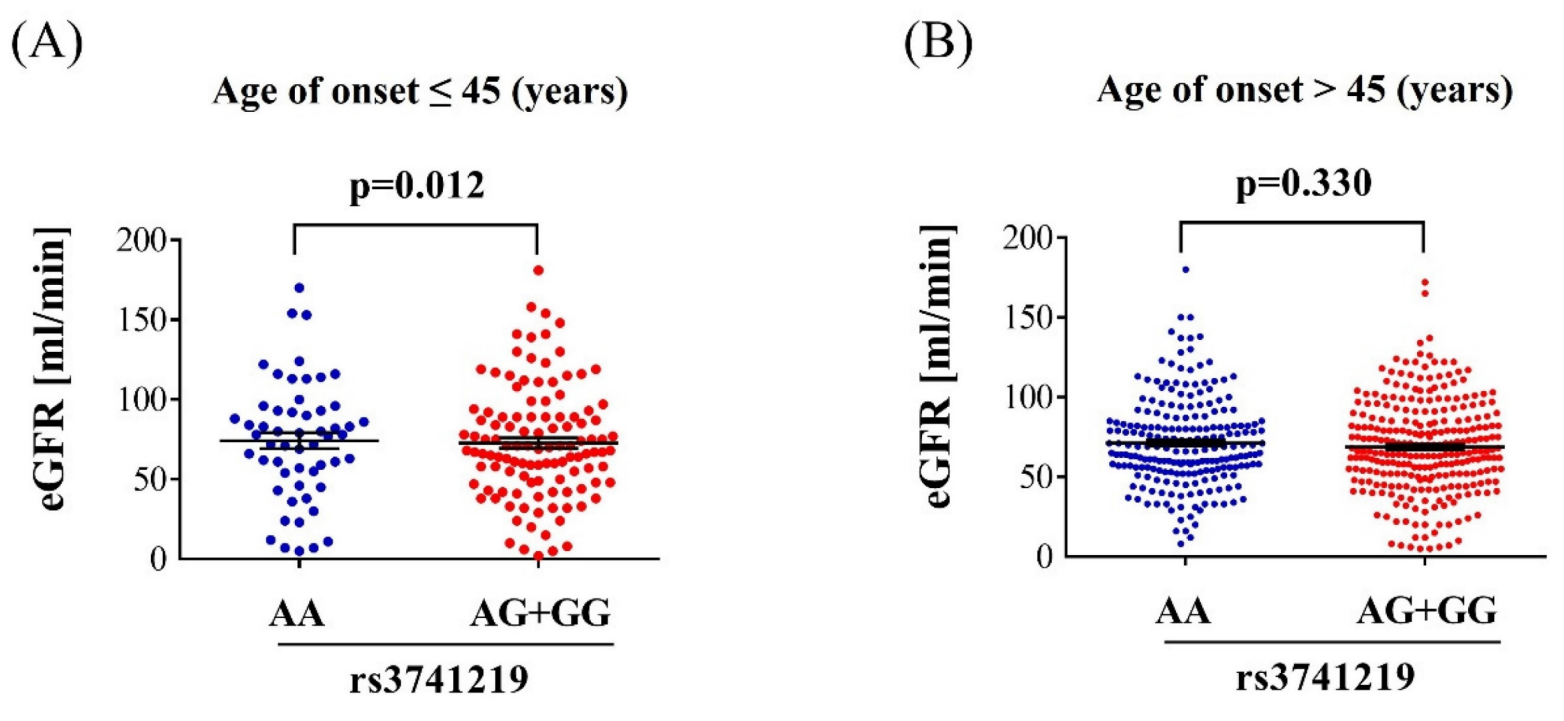
** The glomerular filtration rate in different H19 SNP rs3741219 genotypes.** (A) The glomerular filtration rate in the population with diabetes onset before 45 years. (B) The glomerular filtration rate in the population with diabetes onset after 45 years.

Regarding the clinical characteristics of individuals with diabetes and different *H19* SNP rs3741219 genotypes, those with the *H19* SNP rs3741219 AG+GG genotype was associated with significantly higher serum creatinine levels (p = 0.034), lower glomerular filtration rate (GFR) (p = 0.013), higher total cholesterol/HDL ratio (p = 0.031), and elevated triglyceride levels (p = 0.012) (Table 4). No significant associations were observed between the *H19* SNP rs3741219 genotypes and other clinical characteristics (all p > 0.05) (Table 4). In the subgroup of individuals with diabetes onset before the age of 45, GFR was significantly lower in those with the H19 SNP rs3741219 AG+GG genotype compared to those with the AA genotype (72.66 ± 35.19 versus 84.59 ± 34.84, p = 0.012) (Figure 1A). In contrast, no significant association between GFR and H19 SNP rs3741219 genotypes was observed in individuals with diabetes onset after the age of 45 (68.85 ± 29.07 versus 71.37 ± 27.89, p = 0.330) (Figure 1B).

## Discussion

In the present study, a significant association was identified between the *H19* SNP rs3741219 variant and the risk of DR development in patients with diabetes onset before the age of 45. Furthermore, the *H19* SNP rs3741219 variant was associated with an increased risk of dyslipidemia, elevated serum creatinine levels, and decreased GFR. Notably, decreased GFR was more commonly observed in individuals with diabetes onset before the age of 45 who possessed the *H19* SNP rs3741219 variant genotypes.

The development of DR is influenced by several biochemical and genetic factors, as demonstrated in earlier studies 11, 27, 28. The hyperglycemic status is the basic component for DR development and the HbA1c can predicted the development of DR 5, 9, 29. Additionally, inflammatory cytokines, including members of the interleukin family, VEGF, and tumor necrosis factor-alpha (TNF-α), are significantly elevated in individuals with diabetes and DR 11. Regarding genetic factors, several loci have been linked to an increased risk of DR. Specifically, genetic variants at the PPARG and KCNJ11 loci are associated with a higher incidence of DR development 6. Furthermore, MEG3 methylation has been shown to accelerate endothelial-mesenchymal transition, a critical process in DR formation 30. Under hyperglycemic conditions, MEG3 expression in the human retina is reduced, suggesting that elevated MEG3 levels may have potential therapeutic implications for DR management 31. The ALR2 gene, which regulates the polyol pathway, has also been implicated in DR pathogenesis 29. With regard to genetic polymorphisms and DR, several SNPs have been identified as risk factors for DR. The *SELP* gene variants rs6128, rs6133, and rs3917779 have been associated with a significantly increased risk of DR development 27. In addition, SNPs in the *MMP* gene family, particularly the *MMP-2* SNP rs243864 variant, are significantly more prevalent in the DR population 32, 33. The *HOTAIR* SNP rs12427129 has also been shown to contribute to a higher risk of proliferative DR in Asian populations 34. On the other hand, the H19 gene has been implicated in the development of several disorders, including endometriosis and various malignancies 35-37. The H19 gene is also associated with chronic inflammatory diseases 20, and lower expression of H19 may mitigate hyperglycemia-induced inflammation 38. In terms of genetic polymorphisms, the *H19* SNP rs2839698 variant has been significantly associated with an increased risk of hepatocellular carcinoma 39, and the *H19* SNP rs217727 has been linked to a higher risk of breast cancer development 40. Given that the *H19* gene is involved in the pathophysiology of DR and that its genetic polymorphisms can influence the development of various diseases 20, 41, we hypothesize that *H19* SNPs may also play a role in DR development in specific populations. This hypothesis is partially supported by the findings of the present study.

In relation to the association between *H19* gene SNPs and the presence of DR in the diabetic population, the overall group analysis did not reveal a significant correlation. However, the *H19* SNP rs3741219 variant was associated with an increased risk of DR development in patients with diabetes onset before the age of 45. Previous studies have shown a higher prevalence of DR in individuals with MTHFR gene variants 27, and the *RAGE* gene SNP rs2070600 has also been significantly correlated with DR development 29. Nevertheless, the association between *H19* gene SNPs and DR has not been fully elucidated. To the best of our knowledge, the findings of the present study provide preliminary evidence supporting a significant correlation between the *H19* SNP rs3741219 variant and DR in younger diabetic patients (i.e., those under 45 years old). Furthermore, all participants were regularly followed up in the ophthalmology department by a single ophthalmologist, ensuring consistency in the diagnostic criteria for DR. Additionally, we controlled for potential confounding factors, such as age, sex, HbA1c levels, and other laboratory parameters, in the multiple logistic regression model. As a result, the *H19* SNP rs3741219 variant may serve as an independent predictor for the development of DR in individuals with diabetes onset before the age of 45. Previous research has indicated that younger diabetic patients may experience more rapid progression of DR 42. Moreover, diabetes patients under the age of 40 have been shown to have a fivefold increased risk of developing DR compared to those over the age of 60, after adjusting for potential confounders 43. On the other hand, the *H19* SNP rs3741219 variant has been linked to an increased risk of diseases such as breast cancer 40. Therefore, since the H19 gene influences DR-related factors, including inflammatory and vascular components 38, 44, specific *H19* gene polymorphisms may have a distinct impact on the incidence of DR in younger diabetic patients, who typically present with more severe baseline diabetes status compared to their older counterparts.

Regarding the association between clinical characteristics in the diabetic population and polymorphisms in the *H19* gene, we found that reduced renal function and dyslipidemia were significantly correlated with the *H19* SNP rs3741219 variant. Consistent with earlier studies, the *H19* gene has been shown to influence the development of renal fibrosis in diabetic mice 45. Additionally, the H19 gene is associated with an increased incidence of renal tubular epithelial cell injury 46 , and a higher risk of diabetic kidney disease has been observed in patients with elevated H19 gene expression 47. LncRNA H19 expression has also been linked to a decreased GFR in patients with chronic kidney disease 48. Given that the H19 gene contributes to worsening renal conditions, it is plausible that polymorphisms in the H19 gene could exacerbate renal dysfunction, leading to elevated serum creatinine levels and decreased GFR. In terms of lipid metabolism, the presence of the *H19* SNP rs3741219 variant was associated with elevated serum lipids. Previous studies have shown that the H19 gene regulates lipid droplet metabolism in hepatic cells 49. Furthermore, the H19 gene expression is involved in the regulation of atherosclerosis, a condition for which dyslipidemia is a known predictor 50. Thus, the *H19* SNP rs3741219 variant may contribute to higher serum lipid levels.

In the subgroup analysis stratified by age, reduced GFR was predominantly observed in the diabetic population diagnosed before the age of 45. This phenomenon has been rarely reported in the literature. As discussed earlier, younger individuals with diabetes tend to experience more rapid progression of DR 42, and the deterioration of renal function may be more pronounced in this group under the influence of the *H19* SNP rs3741219 genotype. These findings align with previous research, demonstrating that the *H19* SNP rs3741219 variant is associated with poorer health outcomes in the early-onset diabetes population. However, the precise mechanisms by which the *H19* SNP rs3741219 variant contributes to these conditions require further investigation.

From an epidemiological perspective, diabetes is a widespread disease, affecting approximately four million people in China 51. Among its complications, DR is the most common microvascular complication and affects a significant proportion of the diabetic population 6, 52. In its advanced or proliferative form, DR can lead to intraocular hemorrhage, retinal detachment, and glaucoma, potentially resulting in permanent visual loss 5. According to previous studies, advanced DR accounts for approximately 2.6% of cases of irreversible blindness worldwide 6. Furthermore, diabetic kidney disease (DKD) also affects a large proportion of individuals with diabetes 53, and end-stage renal disease, which requires hemodialysis, is not uncommon in this population 54. Therefore, the management of diabetic kidney disease is of paramount importance. Given the substantial economic burden associated with both DR and diabetic kidney disease, identifying genetic markers for these conditions is essential for better prevention and management strategies.

There are several limitations to the present study. First, the case-control design employed in this study precludes the evaluation of the relationship between *H19* gene polymorphisms and the progression of DR, which would be more appropriately assessed in a cohort design. Additionally, several baseline laboratory parameters in the DR group were significantly worse compared to those in the non-DR group. While genetic polymorphisms are not influenced by these parameters and we controlled for them in the multiple logistic regression model, the substantial differences in baseline characteristics may still introduce potential bias. Moreover, inflammatory biomarkers, such as interleukins and tumor necrosis factor, were not measured in the present study, which could have influenced the results of our statistical analysis. Finally, all participants enrolled in this study were Han Taiwanese, which may limit the generalizability of the findings to other populations, thus reducing the external validity of the study.

In conclusion, the presence of the *H19* SNP rs3741219 variant is associated with a higher risk of DR development in individuals with diabetes onset before the age of 45. Furthermore, the *H19* SNP rs3741219 variant is also linked to reduced renal function, particularly in those with early-onset diabetes. Therefore, ophthalmic and nephrology referrals may be recommended for individuals with the *H19* SNP rs3741219 variant and diabetes onset before 45 years. Future large-scale prospective clinical trials are necessary to further investigate the relationship between *H19* gene SNPs and the therapeutic outcomes of DR.

## Figures and Tables

**Table 1:** Clinical and laboratory characteristics of patients with diabetic retinopathy and no diabetic retinopathy.

Variable	Non-DR group (N=454)	DR group (N=272)	p value
Age (years)	60.22 ± 11.22	62.59 ± 10.79	0.005*
Age of onset (years)	50.84 ± 10.78	50.59 ± 11.17	0.767
Male gender [n (%)]	240 (52.9%)	150 (55.1%)	0.550
Duration of diabetes (years)	9.38 ± 7.03	12.00 ± 7.98	<0.001*
HbA1c [% (mmol/mol)]	6.96 ± 0.99	7.58 ± 1.42	<0.001*
Serum creatinine [mg/dL]	0.89 ± 0.35	1.56 ± 1.85	<0.001*
GFR [ml/min]	78.44 ± 27.69	62.49 ± 33.95	<0.001*
Total cholesterol [mmol/L]	160.52 ± 43.04	165.38 ± 47.65	0.165
HDL cholesterol [μmol/L]	46.29 ± 12.65	43.65 ± 13.45	0.009*
Total cholesterol/HDL ratio	3.70 ± 2.08	4.06 ± 1.51	0.016*
LDL cholesterol [μmol/L]	86.60 ± 28.22	86.44 ± 32.78	0.948
Triglycerides [μmol/L]	140.19 ± 164.95	158.08 ± 118.39	0.127

DR: diabetic retinopathy, GFR: glomerular filtration rate, HbA1c: glycated hemoglobin, N: number * denotes significant difference between groups

**Table 2:** Odds ratio and 95% confidence interval of diabetic retinopathy associated with *H19* genotypic frequencies.

Variable	Non-DR group (N=454)	DR group (N=272)	AOR (95% CI)	p value
**rs3024270**				
CC	125 (27.5%)	79 (29.0%)	1.000	
CG	219 (48.2%)	138 (50.7%)	0.858 (0.577-1.275)	0.448
GG	110 (24.3%)	55 (20.3%)	0.795 (0.493-1.280)	0.345
CG+GG	329 (72.5%)	193 (71.0%)	0.915 (0.760-1.102)	0.349
**rs2839698**				
CC	212 (46.7%)	127 (46.7%)	1.000	
CT	196 (43.2%)	117 (43.0%)	0.829 (0.578-1.189)	0.308
TT	46 (10.1%)	28 (10.3%)	1.084 (0.611-1.924)	0.783
CT+TT	242 (53.3%)	145 (53.3%)	0.935 (0.788-1.108)	0.438
**rs3741219**				
AA	206 (45.4%)	122 (44.9%)	1.000	
AG	200 (44.1%)	121 (44.5%)	0.819 (0.571-1.176)	0.279
GG	48 (10.5%)	29 (10.6%)	1.047 (0.592-1.851)	0.875
AG+GG	248 (54.6%)	150 (55.1%)	0.927 (0.781-1.100)	0.386
**rs2107425**				
CC	170 (37.4%)	102 (37.5%)	1.000	
CT	213 (46.9%)	128 (47.1%)	0.860 (0.592-1.249)	0.428
TT	71 (15.6%)	42 (15.4%)	1.084 (0.653-1.799)	0.754
CT+TT	284 (62.6%)	170 (62.5%)	0.955 (0.802-1.138)	0.605
**rs217727**				
CC	192 (42.3%)	119 (43.8%)	1.000	
CT	206 (45.4%)	122 (44.9%)	0.909 (0.632-1.307)	0.605
TT	56 (12.3%)	31 (11.4%)	1.052 (0.609-1.814)	0.857
CT+TT	262 (57.7%)	153 (56.3%)	0.968 (0.816-1.150)	0.713

AOR: adjusted odds ratio, CI: confidence intervals, N: number The adjusted odds ratio (AOR) with their 95% confidence intervals were estimated by multiple logistic regression models after controlling for age, the duration of diabetes, HbA1c, serum creatinine levels, glomerular filtration rate, HDL cholesterol levels and total cholesterol/HDL ratio.

**Table 3:** Odds ratio and 95% confidence interval of *H19* genotypic frequencies and diabetic retinopathy with different onset age.

Variable	Age of onset ≤ 45 (years) (N=223)			Age of onset >45 (years) (N=503)		
	Non-DR group (N=132)	DR group (N=91)	p value	Non-DR group (N=322)	DR group (N=181)	p value
**rs3024270**						
CC	43 (32.6%)	28 (30.8%)		82 (25.5%)	51 (28.2%)	
CG	60 (45.4%)	46 (50.5%)	0.601	159 (49.4%)	92 (50.8%)	0.744
GG	29 (22.0%)	17 (18.7%)	0.788	81 (25.1%)	38 (21.0%)	0.288
CG+GG	89 (67.4%)	63 (69.2%)	0.776	240 (74.5%)	130 (71.8%)	0.508
**rs2839698**						
CC	71 (53.8%)	41 (45.1%)		141 (43.8%)	86 (47.5%)	
CT	47 (35.6%)	40 (44.0%)	0.183	149 (46.3%)	77 (42.5%)	0.398
TT	14 (10.6%)	10 (11.0%)	0.643	32 (9.9%)	18 (10.0%)	0.803
CT+TT	61 (46.2%)	50 (54.9%)	0.200	181 (56.2%)	95 (52.5%)	0.420
**rs3741219**						
AA	71 (53.8%)	36 (39.6%)		135 (41.9%)	86 (47.5%)	
AG	47 (35.6%)	45 (49.4%)	0.030*^,a^	153 (47.5%)	76 (42.0%)	0.206
GG	14 (10.6%)	10 (11.0%)	0.458	34 (10.6%)	19 (10.5%)	0.680
AG+GG	61 (46.2%)	55 (60.4%)	0.037*^,b^	187 (58.1%)	95 (52.5%)	0.226
**rs2107425**						
CC	49 (37.1%)	29 (31.9%)		121 (37.6%)	73 (40.3%)	
CT	53 (40.2%)	44 (48.4%)	0.276	160 (49.7%)	84 (46.4%)	0.488
TT	30 (22.7%)	18 (19.7%)	0.971	41 (12.7%)	24 (13.3%)	0.919
CT+TT	83 (62.9%)	62 (68.1%)	0.419	201 (62.4%)	108 (59.7%)	0.543
**rs217727**						
CC	54 (40.9%)	36 (49.6%)		138 (42.9%)	83 (45.9%)	
CT	55 (41.7%)	41 (45.1%)	0.708	151 (46.9%)	81 (44.8%)	0.559
TT	23 (17.4%)	14 (15.4%)	0.821	33 (10.2%)	17 (9.4%)	0.638
CT+TT	78 (59.1%)	55 (60.4%)	0.840	184 (57.1%)	98 (54.1%)	0.515

DR: diabetic retinopathy, N: number * denotes significant difference between groups ^a^OR (95% CI): 1.888 (1.065-3.348) ^b^OR (95% CI): 1.778 (1.034-3.057)

**Table 4:** Clinical characteristics of diabetes patients according to *H19* rs3741219 genotypes.

Variable^#^	*H19* rs3741219		
	AA (n=328)	AG+GG (n=398)	p value
Duration of diabetes (years)	10.11 ± 7.56	10.57 ± 7.45	0.411
HbA1c [% (mmol/mol)]	7.19 ± 1.26	7.19 ± 1.15	0.990
Serum creatinine [mg/dL]	1.03 ± 0.86	1.22 ± 1.41	0.034*
GFR [ml/min]	75.74 ± 30.94	69.97 ± 31.01	0.013*
Total cholesterol [mmol/L]	160.06 ± 37.86	164.10 ± 49.78	0.231
HDL cholesterol [μmol/L]	46.37 ± 13.33	44.49 ± 12.66	0.055
Total cholesterol/HDL ratio	3.66 ± 1.19	3.97 ± 2.33	0.031*
LDL cholesterol [μmol/L]	86.53 ± 28.41	86.55 ± 31.16	0.995
Triglycerides [μmol/L]	131.11 ± 92.84	159.53 ± 183.58	0.012*

^#^ Quantitative data are represented as means ± standard deviation. DR: diabetic retinopathy, GFR: glomerular filtration rate, HbA1c: glycated hemoglobin, N: number * denotes significant difference between group
